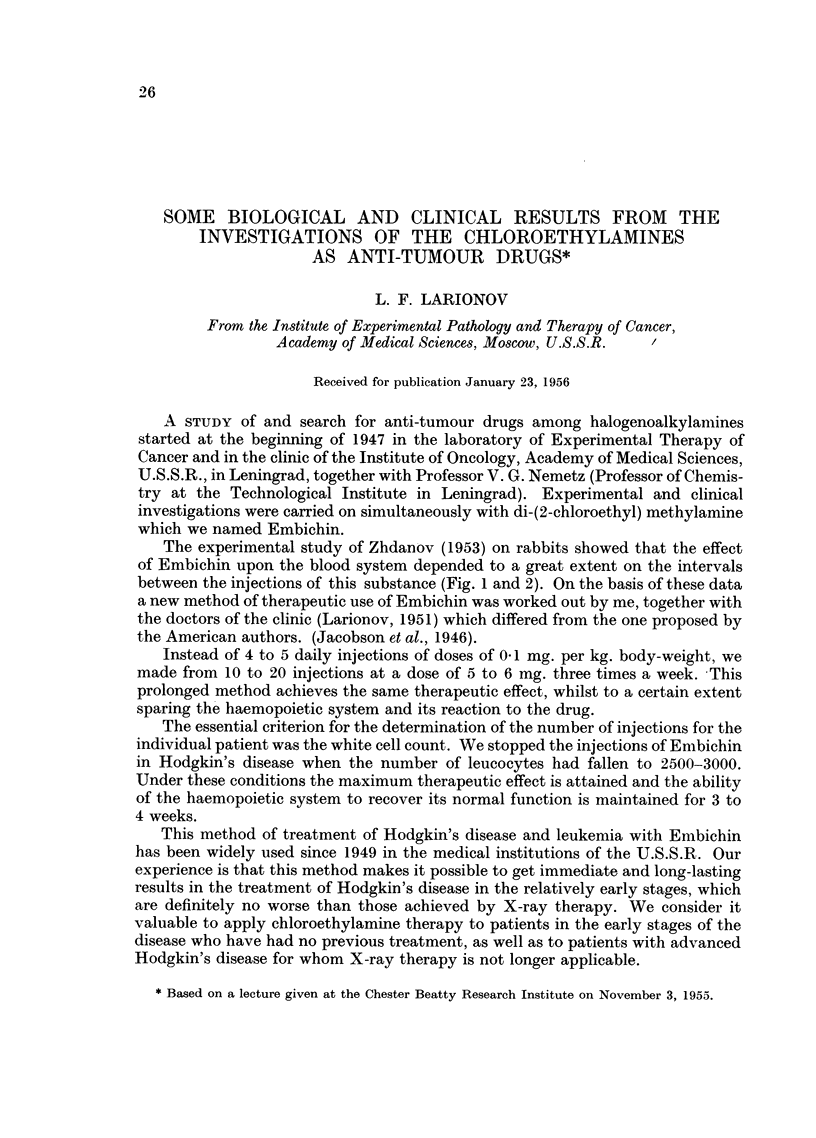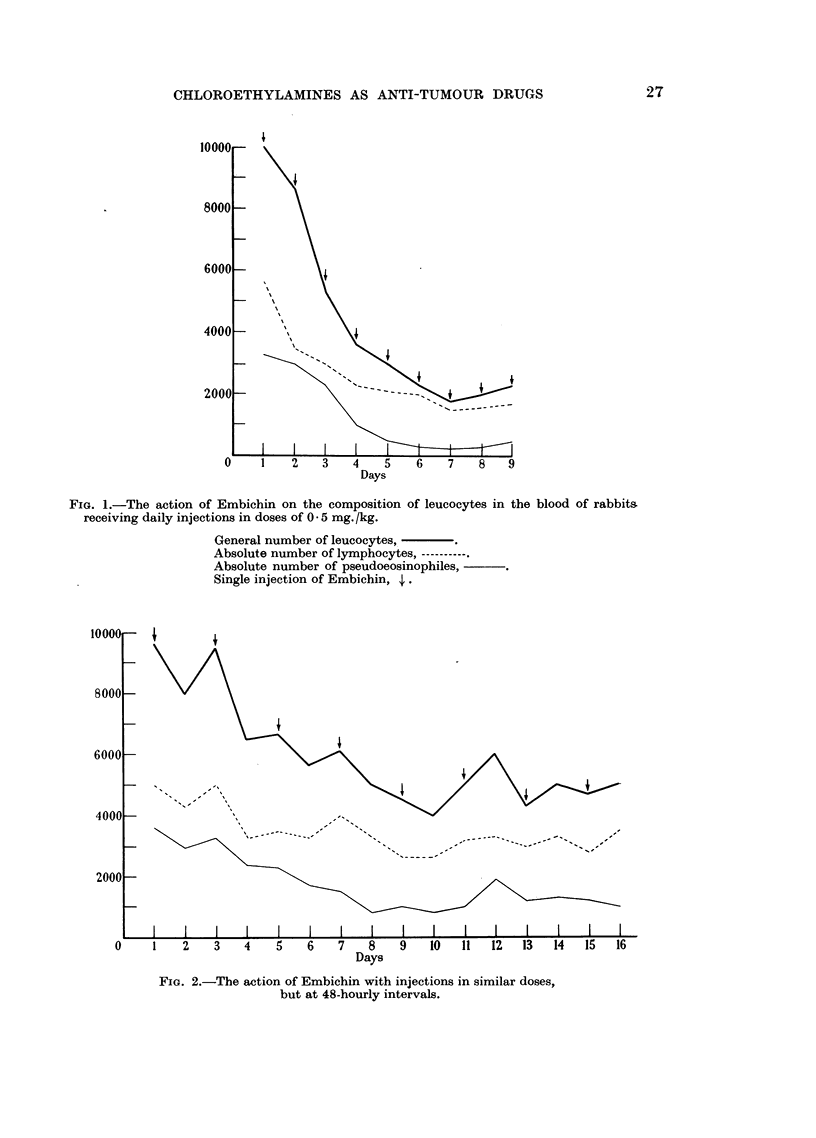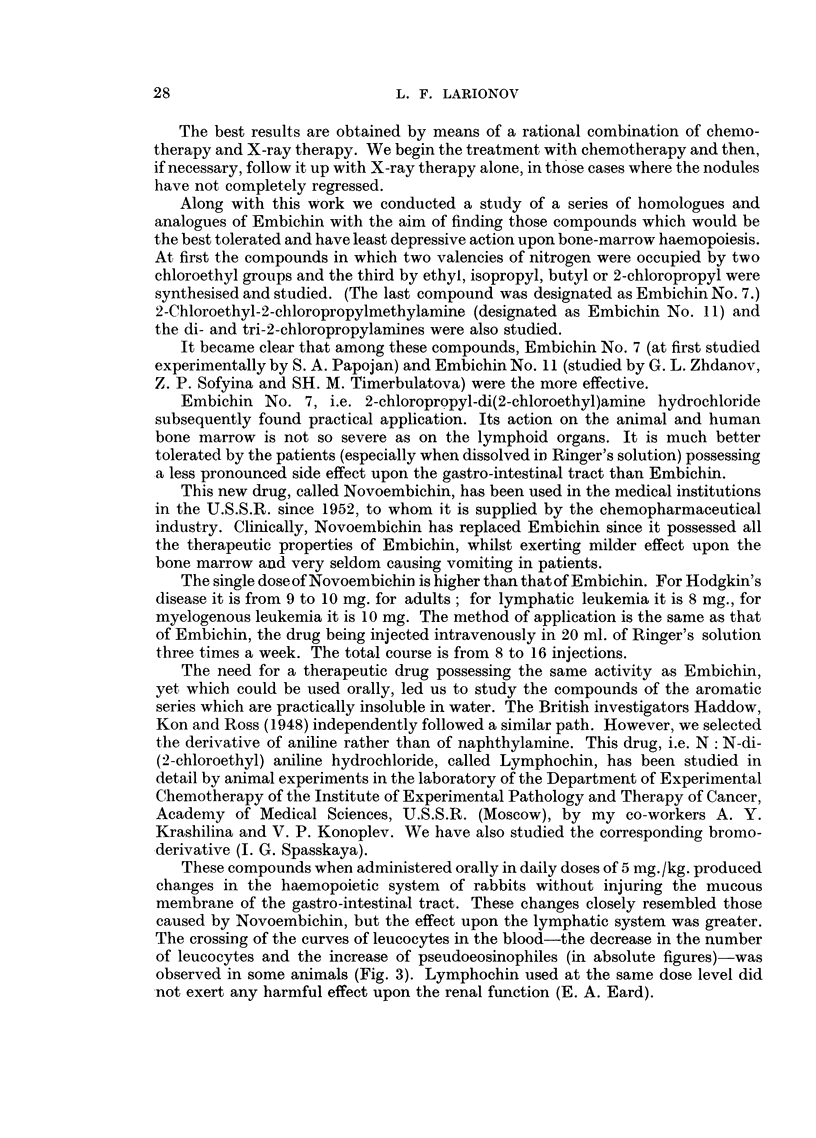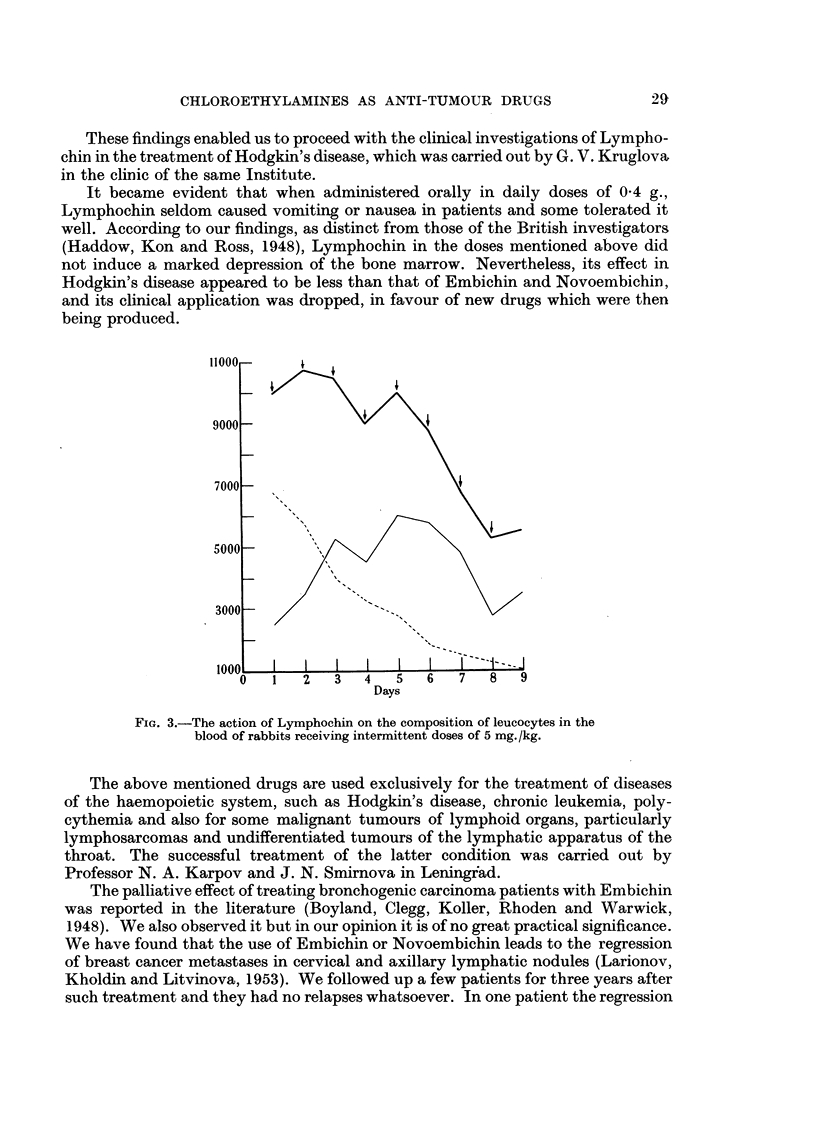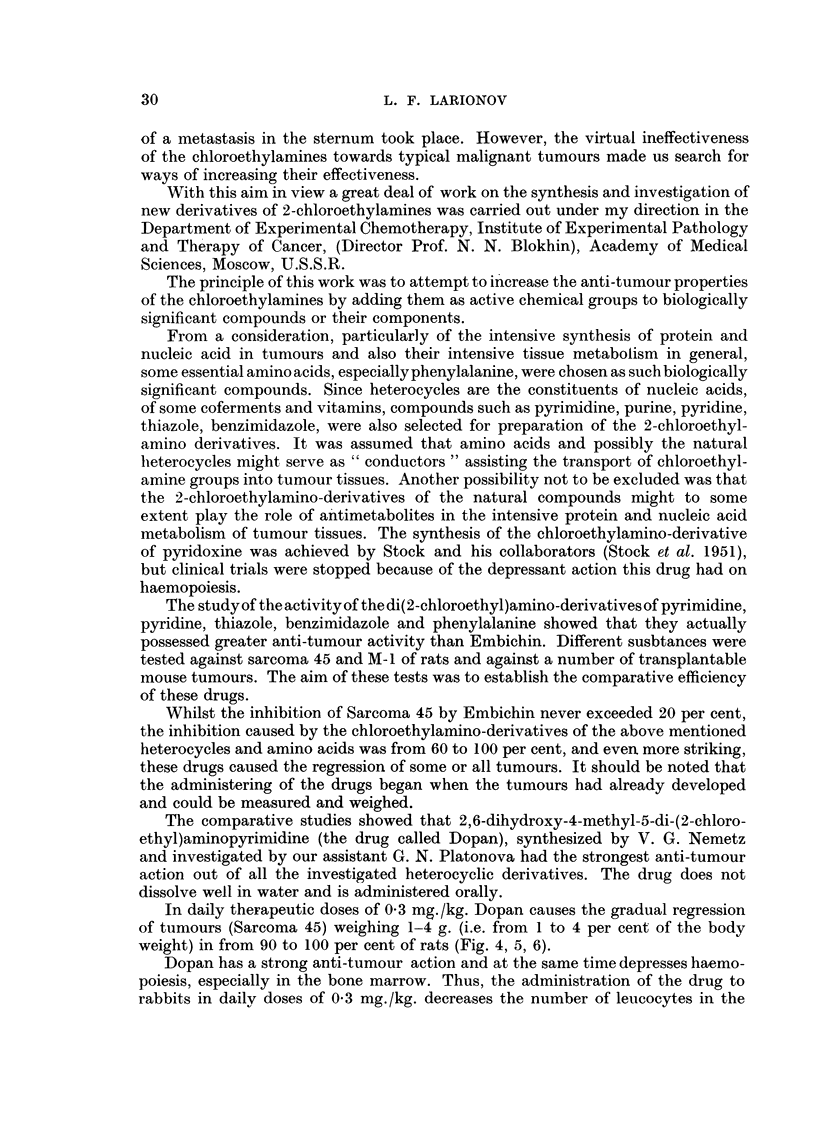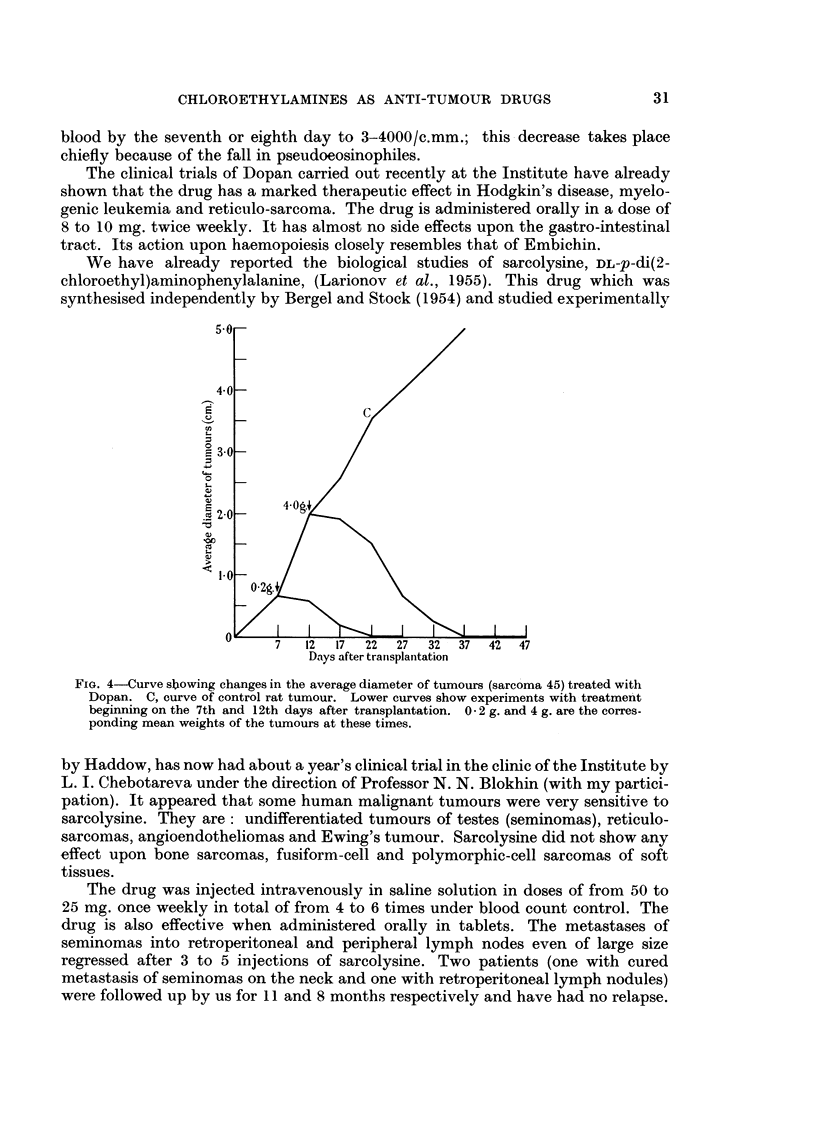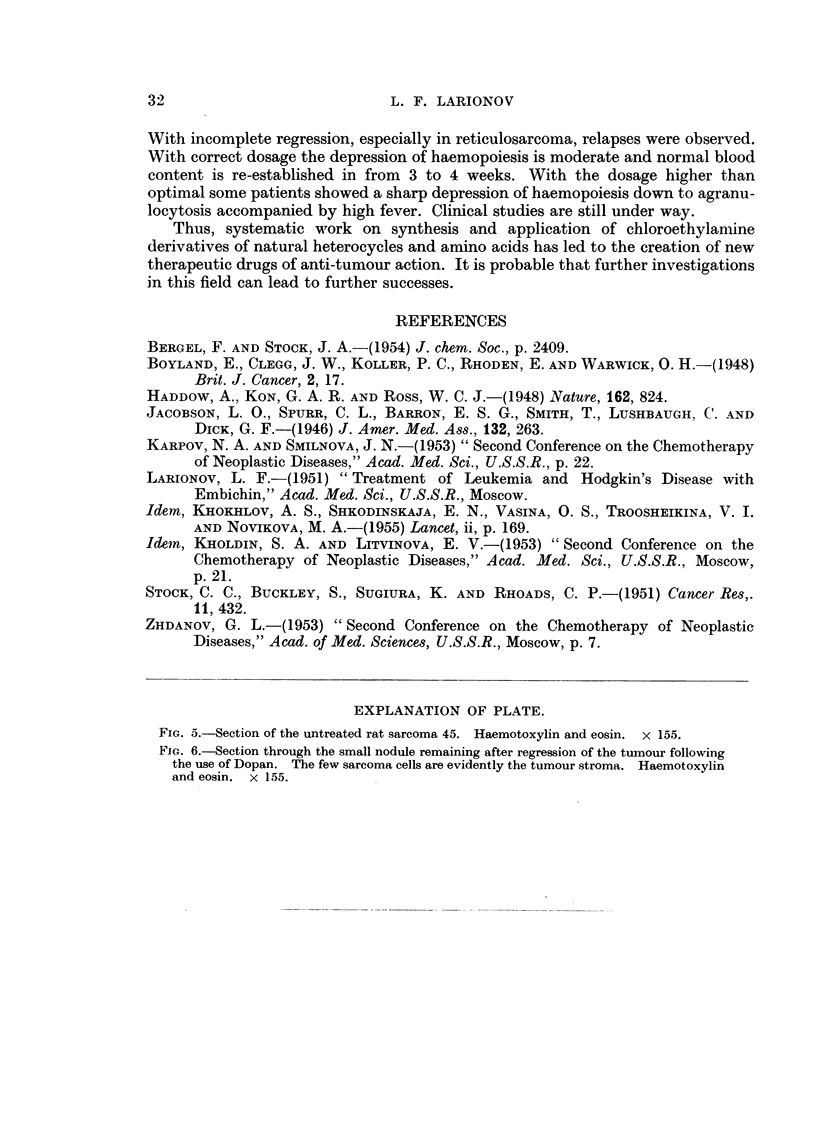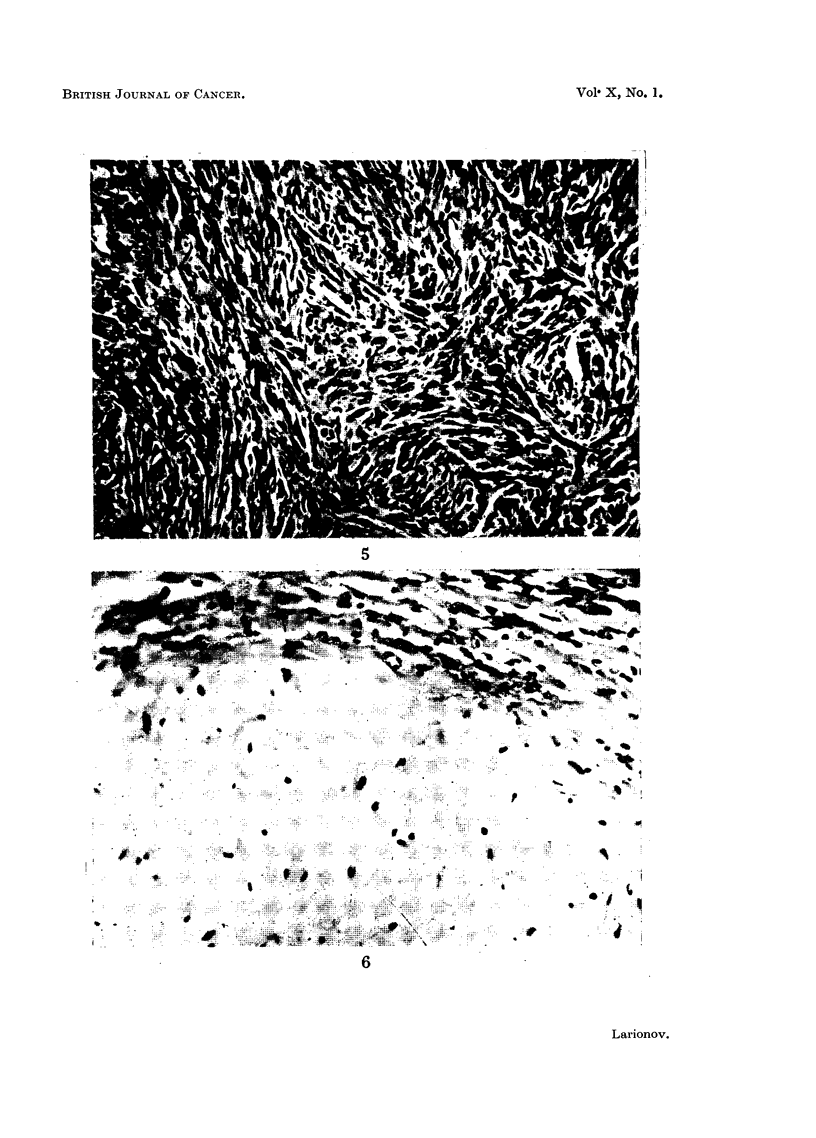# Some Biological and Clinical Results from the Investigations of the Chloroethylamines as Anti-Tumour Drugs*

**DOI:** 10.1038/bjc.1956.4

**Published:** 1956-03

**Authors:** L. F. Larionov

## Abstract

**Images:**


### SOME BIOLOGICAL AND CLINICAL RESULTS FROM THE INVESTIGATIONS OF THE CHLOROETHYLAMINES AS ANTI-TUMOUR DRUGS*

L. F. LARIONOV

*From the Institute of Experimental Pathology and Therapy of Cancer, Academy of Medical Sciences, Moscow, U.S.S.R.*

Received for publication January 23, 1956

A STUDY of and search for anti-tumour drugs among halogenoalkylamines started at the beginning of 1947 in the laboratory of Experimental Therapy of Cancer and in the clinic of the Institute of Oncology, Academy of Medical Sciences, U.S.S.R., in Leningrad, together with Professor V. G. Nemetz (Professor of Chemistry at the Technological Institute in Leningrad). Experimental and clinical investigations were carried on simultaneously with di-(2-chloroethyl) methylamine which we named Embichin.

The experimental study of Zhdanov (1953) on rabbits showed that the effect of Embichin upon the blood system depended to a great extent on the intervals between the injections of this substance (Fig. 1 and 2). On the basis of these data a new method of therapeutic use of Embichin was worked out by me, together with the doctors of the clinic (Larionov, 1951) which differed from the one proposed by the American authors. (Jacobson *et al.*, 1946).

Instead of 4 to 5 daily injections of doses of 0·1 mg. per kg. body-weight, we made from 10 to 20 injections at a dose of 5 to 6 mg. three times a week. This prolonged method achieves the same therapeutic effect, whilst to a certain extent sparing the haemopoietic system and its reaction to the drug.

The essential criterion for the determination of the number of injections for the individual patient was the white cell count. We stopped the injections of Embichin in Hodgkin's disease when the number of leucocytes had fallen to 2500–3000. Under these conditions the maximum therapeutic effect is attained and the ability of the haemopoietic system to recover its normal function is maintained for 3 to 4 weeks.

This method of treatment of Hodgkin's disease and leukemia with Embichin has been widely used since 1949 in the medical institutions of the U.S.S.R. Our experience is that this method makes it possible to get immediate and long-lasting results in the treatment of Hodgkin's disease in the relatively early stages, which are definitely no worse than those achieved by X-ray therapy. We consider it valuable to apply chloroethylamine therapy to patients in the early stages of the disease who have had no previous treatment, as well as to patients with advanced Hodgkin's disease for whom X-ray therapy is not longer applicable.

* Based on a lecture given at the Chester Beatty Research Institute on November 3, 1955.

FIG. 1.—The action of Embichin on the composition of leucocytes in the blood of rabbits receiving daily injections in doses of 0·5 mg./kg.

General number of leucocytes, ————.
Absolute number of lymphocytes, ----------.
Absolute number of pseudoeosinophiles, ————.
Single injection of Embichin, ↓.

FIG. 2.—The action of Embichin with injections in similar doses, but at 48-hourly intervals.

The best results are obtained by means of a rational combination of chemotherapy and X-ray therapy. We begin the treatment with chemotherapy and then, if necessary, follow it up with X-ray therapy alone, in those cases where the nodules have not completely regressed.

Along with this work we conducted a study of a series of homologues and analogues of Embichin with the aim of finding those compounds which would be the best tolerated and have least depressive action upon bone-marrow haemopoiesis. At first the compounds in which two valencies of nitrogen were occupied by two chloroethyl groups and the third by ethyl, isopropyl, butyl or 2-chloropropyl were synthesised and studied. (The last compound was designated as Embichin No. 7.) 2-Chloroethyl-2-chloropropylmethylamine (designated as Embichin No. 11) and the di- and tri-2-chloropropylamines were also studied.

It became clear that among these compounds, Embichin No. 7 (at first studied experimentally by S. A. Papojan) and Embichin No. 11 (studied by G. L. Zhdanov, Z. P. Sofyina and SH. M. Timerbulatova) were the more effective.

Embichin No. 7, i.e. 2-chloropropyl-di(2-chloroethyl)amine hydrochloride subsequently found practical application. Its action on the animal and human bone marrow is not so severe as on the lymphoid organs. It is much better tolerated by the patients (especially when dissolved in Ringer's solution) possessing a less pronounced side effect upon the gastro-intestinal tract than Embichin.

This new drug, called Novoembichin, has been used in the medical institutions in the U.S.S.R. since 1952, to whom it is supplied by the chemopharmaceutical industry. Clinically, Novoembichin has replaced Embichin since it possessed all the therapeutic properties of Embichin, whilst exerting milder effect upon the bone marrow and very seldom causing vomiting in patients.

The single dose of Novoembichin is higher than that of Embichin. For Hodgkin's disease it is from 9 to 10 mg. for adults ; for lymphatic leukemia it is 8 mg., for myelogenous leukemia it is 10 mg. The method of application is the same as that of Embichin, the drug being injected intravenously in 20 ml. of Ringer's solution three times a week. The total course is from 8 to 16 injections.

The need for a therapeutic drug possessing the same activity as Embichin, yet which could be used orally, led us to study the compounds of the aromatic series which are practically insoluble in water. The British investigators Haddow, Kon and Ross (1948) independently followed a similar path. However, we selected the derivative of aniline rather than of naphthylamine. This drug, i.e. N : N-di-(2-chloroethyl) aniline hydrochloride, called Lymphochin, has been studied in detail by animal experiments in the laboratory of the Department of Experimental Chemotherapy of the Institute of Experimental Pathology and Therapy of Cancer, Academy of Medical Sciences, U.S.S.R. (Moscow), by my co-workers A. Y. Krashilina and V. P. Konoplev. We have also studied the corresponding bromo-derivative (I. G. Spasskaya).

These compounds when administered orally in daily doses of 5 mg./kg. produced changes in the haemopoietic system of rabbits without injuring the mucous membrane of the gastro-intestinal tract. These changes closely resembled those caused by Novoembichin, but the effect upon the lymphatic system was greater. The crossing of the curves of leucocytes in the blood—the decrease in the number of leucocytes and the increase of pseudoeosinophiles (in absolute figures)—was observed in some animals (Fig. 3). Lymphochin used at the same dose level did not exert any harmful effect upon the renal function (E. A. Eard).

These findings enabled us to proceed with the clinical investigations of Lymphochin in the treatment of Hodgkin's disease, which was carried out by G. V. Kruglova in the clinic of the same Institute.

It became evident that when administered orally in daily doses of 0·4 g., Lymphochin seldom caused vomiting or nausea in patients and some tolerated it well. According to our findings, as distinct from those of the British investigators (Haddow, Kon and Ross, 1948), Lymphochin in the doses mentioned above did not induce a marked depression of the bone marrow. Nevertheless, its effect in Hodgkin's disease appeared to be less than that of Embichin and Novoembichin, and its clinical application was dropped, in favour of new drugs which were then being produced.

FIG. 3.—The action of Lymphochin on the composition of leucocytes in the blood of rabbits receiving intermittent doses of 5 mg./kg.

The above mentioned drugs are used exclusively for the treatment of diseases of the haemopoietic system, such as Hodgkin's disease, chronic leukemia, polycythemia and also for some malignant tumours of lymphoid organs, particularly lymphosarcomas and undifferentiated tumours of the lymphatic apparatus of the throat. The successful treatment of the latter condition was carried out by Professor N. A. Karpov and J. N. Smirnova in Leningrad.

The palliative effect of treating bronchogenic carcinoma patients with Embichin was reported in the literature (Boyland, Clegg, Koller, Rhoden and Warwick, 1948). We also observed it but in our opinion it is of no great practical significance. We have found that the use of Embichin or Novoembichin leads to the regression of breast cancer metastases in cervical and axillary lymphatic nodules (Larionov, Kholdin and Litvinova, 1953). We followed up a few patients for three years after such treatment and they had no relapses whatsoever. In one patient the regression of a metastasis in the sternum took place. However, the virtual ineffectiveness of the chloroethylamines towards typical malignant tumours made us search for ways of increasing their effectiveness.

With this aim in view a great deal of work on the synthesis and investigation of new derivatives of 2-chloroethylamines was carried out under my direction in the Department of Experimental Chemotherapy, Institute of Experimental Pathology and Therapy of Cancer, (Director Prof. N. N. Blokhin), Academy of Medical Sciences, Moscow, U.S.S.R.

The principle of this work was to attempt to increase the anti-tumour properties of the chloroethylamines by adding them as active chemical groups to biologically significant compounds or their components.

From a consideration, particularly of the intensive synthesis of protein and nucleic acid in tumours and also their intensive tissue metabolism in general, some essential amino acids, especially phenylalanine, were chosen as such biologically significant compounds. Since heterocycles are the constituents of nucleic acids, of some coferments and vitamins, compounds such as pyrimidine, purine, pyridine, thiazole, benzimidazole, were also selected for preparation of the 2-chloroethylamino derivatives. It was assumed that amino acids and possibly the natural heterocycles might serve as " conductors " assisting the transport of chloroethylamine groups into tumour tissues. Another possibility not to be excluded was that the 2-chloroethylamino-derivatives of the natural compounds might to some extent play the role of antimetabolites in the intensive protein and nucleic acid metabolism of tumour tissues. The synthesis of the chloroethylamino-derivative of pyridoxine was achieved by Stock and his collaborators (Stock *et al.* 1951), but clinical trials were stopped because of the depressant action this drug had on haemopoiesis.

The study of the activity of the di(2-chloroethyl)amino-derivatives of pyrimidine, pyridine, thiazole, benzimidazole and phenylalanine showed that they actually possessed greater anti-tumour activity than Embichin. Different susbtances were tested against sarcoma 45 and M-1 of rats and against a number of transplantable mouse tumours. The aim of these tests was to establish the comparative efficiency of these drugs.

Whilst the inhibition of Sarcoma 45 by Embichin never exceeded 20 per cent, the inhibition caused by the chloroethylamino-derivatives of the above mentioned heterocycles and amino acids was from 60 to 100 per cent, and even more striking, these drugs caused the regression of some or all tumours. It should be noted that the administering of the drugs began when the tumours had already developed and could be measured and weighed.

The comparative studies showed that 2,6-dihydroxy-4-methyl-5-di-(2-chloroethyl)aminopyrimidine (the drug called Dopan), synthesized by V. G. Nemetz and investigated by our assistant G. N. Platonova had the strongest anti-tumour action out of all the investigated heterocyclic derivatives. The drug does not dissolve well in water and is administered orally.

In daily therapeutic doses of 0·3 mg./kg. Dopan causes the gradual regression of tumours (Sarcoma 45) weighing 1–4 g. (i.e. from 1 to 4 per cent of the body weight) in from 90 to 100 per cent of rats (Fig. 4, 5, 6).

Dopan has a strong anti-tumour action and at the same time depresses haemopoiesis, especially in the bone marrow. Thus, the administration of the drug to rabbits in daily doses of 0·3 mg./kg. decreases the number of leucocytes in the blood by the seventh or eighth day to 3–4000/c.mm.; this decrease takes place chiefly because of the fall in pseudoeosinophiles.

The clinical trials of Dopan carried out recently at the Institute have already shown that the drug has a marked therapeutic effect in Hodgkin's disease, myelogenic leukemia and reticulo-sarcoma. The drug is administered orally in a dose of 8 to 10 mg. twice weekly. It has almost no side effects upon the gastro-intestinal tract. Its action upon haemopoiesis closely resembles that of Embichin.

We have already reported the biological studies of sarcolysine, DL-*p*-di(2-chloroethyl)aminophenylalanine, (Larionov *et al.*, 1955). This drug which was synthesised independently by Bergel and Stock (1954) and studied experimentally

FIG. 4—Curve showing changes in the average diameter of tumours (sarcoma 45) treated with Dopan. C, curve of control rat tumour. Lower curves show experiments with treatment beginning on the 7th and 12th days after transplantation. 0·2 g. and 4 g. are the corresponding mean weights of the tumours at these times.

by Haddow, has now had about a year's clinical trial in the clinic of the Institute by L. I. Chebotareva under the direction of Professor N. N. Blokhin (with my participation). It appeared that some human malignant tumours were very sensitive to sarcolysine. They are : undifferentiated tumours of testes (seminomas), reticulo-sarcomas, angioendotheliomas and Ewing's tumour. Sarcolysine did not show any effect upon bone sarcomas, fusiform-cell and polymorphic-cell sarcomas of soft tissues.

The drug was injected intravenously in saline solution in doses of from 50 to 25 mg. once weekly in total of from 4 to 6 times under blood count control. The drug is also effective when administered orally in tablets. The metastases of seminomas into retroperitoneal and peripheral lymph nodes even of large size regressed after 3 to 5 injections of sarcolysine. Two patients (one with cured metastasis of seminomas on the neck and one with retroperitoneal lymph nodules) were followed up by us for 11 and 8 months respectively and have had no relapse.

With incomplete regression, especially in reticulosarcoma, relapses were observed. With correct dosage the depression of haemopoiesis is moderate and normal blood content is re-established in from 3 to 4 weeks. With the dosage higher than optimal some patients showed a sharp depression of haemopoiesis down to agranulocytosis accompanied by high fever. Clinical studies are still under way.

Thus, systematic work on synthesis and application of chloroethylamine derivatives of natural heterocycles and amino acids has led to the creation of new therapeutic drugs of anti-tumour action. It is probable that further investigations in this field can lead to further successes.

### REFERENCES

Bergel, F. and Stock, J. A.—(1954) *J. chem. Soc.*, p. 2409.

Boyland, E., Clegg, J. W., Koller, P. C., Rhoden, E. and Warwick, O. H.—(1948) *Brit. J. Cancer*, **2**, 17.

Haddow, A., Kon, G. A. R. and Ross, W. C. J.—(1948) *Nature*, **162**, 824.

Jacobson, L. O., Spurr, C. L., Barron, E. S. G., Smith, T., Lushbaugh, C. and Dick, G. F.—(1946) *J. Amer. Med. Ass.*, **132**, 263.

Karpov, N. A. and Smilnova, J. N.—(1953) " Second Conference on the Chemotherapy of Neoplastic Diseases," *Acad. Med. Sci., U.S.S.R.*, p. 22.

Larionov, L. F.—(1951) " Treatment of Leukemia and Hodgkin's Disease with Embichin," *Acad. Med. Sci., U.S.S.R.*, Moscow.

*Idem*, Khokhlov, A. S., Shkodinskaja, E. N., Vasina, O. S., Troosheikina, V. I. and Novikova, M. A.—(1955) *Lancet*, ii, p. 169.

*Idem*, Kholdin, S. A. and Litvinova, E. V.—(1953) " Second Conference on the Chemotherapy of Neoplastic Diseases," *Acad. Med. Sci., U.S.S.R.*, Moscow, p. 21.

Stock, C. C., Buckley, S., Sugiura, K. and Rhoads, C. P.—(1951) *Cancer Res,*. **11**, 432.

Zhdanov, G. L.—(1953) " Second Conference on the Chemotherapy of Neoplastic Diseases," *Acad. of Med. Sciences, U.S.S.R.*, Moscow, p. 7.

---

### EXPLANATION OF PLATE.

Fig. 5.—Section of the untreated rat sarcoma 45. Haemotoxylin and eosin. × 155.

Fig. 6.—Section through the small nodule remaining after regression of the tumour following the use of Dopan. The few sarcoma cells are evidently the tumour stroma. Haemotoxylin and eosin. × 155.